# Polyelectrolyte Multilayer-Treated Electrodes for Real-Time Electronic Sensing of Cell Proliferation

**DOI:** 10.6028/jres.115.005

**Published:** 2010-04-01

**Authors:** Geraldine I. Mijares, Darwin R. Reyes, Jon Geist, Michael Gaitan, Brian J. Polk, Don L. DeVoe

**Affiliations:** Semiconductor Electronics Division, National Institute of Standards and Technology, Gaithersburg, MD 20899-8120; Formerly of Semiconductor Electronics Division, National Institute of Standards and Technology, Gaithersburg, MD 20899-8120; Department of Bioengineering and Department of Machanical Engineering, University of Maryland College Park, MD 20742

**Keywords:** biosensor, cell growth, electrical monitoring, gold electrodes, impedance spectroscopy, polyelectrolyte multilayers

## Abstract

We report on the use of polyelectrolyte multilayer (PEM) coatings as a non-biological surface preparation to facilitate uniform cell attachment and growth on patterned thin-film gold (Au) electrodes on glass for impedance-based measurements. Extracellular matrix (ECM) proteins are commonly utilized as cell adhesion promoters for electrodes; however, they exhibit degradation over time, thereby imposing limitations on the duration of conductance-based biosensor experiments. The motivation for the use of PEM coatings arises from their long-term surface stability as promoters for cell attachment, patterning, and culture. In this work, a cell proliferation monitoring device was fabricated. It consisted of thin-film Au electrodes deposited with a titanium-tungsten (TiW) adhesion layer that were patterned on a glass substrate and passivated to create active electrode areas. The electrode surfaces were then treated with a poly(ethyleneimine) (PEI) anchoring layer and subsequent bilayers of sodium poly(styrene sulfonate) (PSS) and poly(allylamine hydrochloride) (PAH). NIH-3T3 mouse embryonic fibroblast cells were cultured on the device, observed by optical microscopy, and showed uniform growth characteristics similar to those observed on a traditional polystyrene cell culture dish. The optical observations were correlated to electrical measurements on the PEM-treated electrodes, which exhibited a rise in impedance with cell proliferation and stabilized to an approximate 15 % increase as the culture approached confluency. In conclusion, cells proliferate uniformly over gold and glass PEM-treated surfaces, making them useful for continuous impedance-based, real-time monitoring of cell proliferation and for the determination of cell growth rate in cellular assays.

## 1. Introduction

Impedance-based biosensors are used to detect cell viability by monitoring cell attachment and surface coverage during cell proliferation [1–3]. If adherent cells are subjected to a stimulus that negatively affects their health and function, it is likely that the cells will begin to detach from the surface on which they are cultured [1,4]. Impedance-based cellular biosensors interface living biological cells with electrodes that serve as a non-invasive, real-time detector for the analysis of cellular responses and physiological changes caused by a chemical, biological, or other type of applied stimuli [3]. In order to detect changes in cell behavior, the impedance is measured as a change in the electrical signal at the interface between an adherent cell and the electrode [1,3]. As a result, this measurement can be sensitive to changes in attachment, spreading, growth, motility, and death, which are all influenced by a variety of stimuli.

Impedance spectroscopy utilizing the electric cell-substrate impedance sensing (ECIS) technology^1^ developed by Giaever and Keese has been used for monitoring the spreading [5,6], motility [7,8], and viability [9,10] of cells by electrical detection. Changes in impedance were correlated to the attachment and motion of cells on electrodes [1,2,11,12], as well as cell shape changes [4]. Since cell cytotoxicity response can exhibit such changes, ECIS has been demonstrated as a powerful tool in cytotoxicity assays for chemical agents [10,13,14] and electroporation/wound healing [15,16] assays. One challenge for electrode-based cell monitoring is the limitation that the passivation layer, which is deposited to cover the electrode and then later removed in specific regions to create the active electrode areas (i.e., where the cells and the medium are in electrical contact with the electrode), typically imposes on the lifetime of the device, as well as the range of solvents chosen for surface preparation and cleaning. Consequently, increasing the stability of passivation layers for cellular impedance electrode configurations is desirable. Furthermore, thin-film planar electrodes that can be patterned on and integrated within microfluidic devices [17–21] are also desirable for applications that require real-time monitoring of cell viability and confluency of cell culture arrays.

Cell attachment and proliferation on the surface of planar electrodes and biosensors, including those utilizing ECIS, are typically promoted using extracellular matrix (ECM) constituents. Examples of ECM constituents, which provide a charged, wettable surface for the promotion of cell adhesion, include fibronectin, collagen, elastin, and laminin. The ECM proteins are physically adsorbed onto the electrode surfaces and induce the secretion of molecules that result in adhesive interactions [22,23]. However, limitations of using matrix proteins include the degradation of biological peptide bonds and possible conformational changes [24] that lead to a shortening of the lifetime of the devices.

One alternative that can address the issue of electrode stability and longevity is to use polyelectrolyte multilayer (PEM) coatings. PEMs are non-biological materials that have been shown to be stable under biological conditions [25–27]. The stability of this material is related to the high charge density and low ionic strength of each polyelectrolyte [28]. PEM coatings are also an excellent choice for modifying a surface to promote cell adhesion because of the ability to control the film thickness and molecular architecture with pH and ionic concentration during the deposition process. The multilayers can be assembled by a simple aqueous process involving the alternate adsorption of polycations and polyanions through electrostatic interactions [29].

PEM coatings are already used in numerous systems including those for in vivo biomedical applications [27], biosensors for immunosensing [30,31], electro-osmotic flow control in microfluidics [32,33], and selective cell patterning [25,26,34]. PEMs can also be functionalized to incorporate biomolecules, such as proteins [30,35] or enzymes [36], into the layers without biomolecular degradation. Additionally, PEMs provide a cost-effective biocompatible electrode coating and a *standardizable* surface that is controlled by the deposition process, producing highly uniform and reproducible films.

PEMs are commonly attached to gold surfaces by using a self-assembled monolayer (SAM) adhesion layer, such as an alkanethiol, to serve as a bridge between gold and the PEMs [30,37–39]. However, the thiol group is rapidly oxidized when SAMs attached to gold are exposed to ambient air conditions, leading to SAM desorption from the gold surface [40,41]. This results in the lifetime of PEMs attached to SAMs on gold surfaces to be less than 24 hours [42]. An alternative way to form a stable PEM coating on a gold electrode that we explore in this paper is to use a polyelectrolyte anchoring layer, such as poly(ethyleneimine) (PEI), which has a strong binding ability from its primary, secondary, and tertiary amine groups onto a number of surfaces [29], including gold. This approach has been shown to be highly stable on negatively-charged substrates, such as surface-treated metal [43], glass [44,45], or silicon [45,46] surfaces and citrate-functionalized gold nanoparticles [45,47]. Therefore, it could also be useful for stabilizing the electrode coatings for cellular impedance measurements, as shown here.

In this report, the fabrication and evaluation of an impedance-based cell proliferation monitoring device combining optically-transparent gold (Au) electrodes with PEM films for cell attachment are presented. The surface coating chemistry that was used was a PEI anchoring layer followed by a number of sodium poly(styrene sulfonate)/poly(allylamine hydrochloride) (PSS/PAH) overlying layers to form an electrode coating consisting solely of PEMs. The working electrode on the device was comprised of nine active electrode areas that were exposed to the medium/cells solution. Experiments were carried out to test PEM-treated electrode surfaces for their suitability to conduct impedance measurements during cell growth, in addition to assessing cell growth homogeneity through the use of the working electrode active area array. The results were compared with optical cell growth measurements on standard polystyrene cell culture surfaces. The array of small active electrode areas has the potential to be fabricated as a series of independent electrodes that could monitor differences in cell behavior throughout the culture chamber. These cell behavior differences can be a result of exposure to a concentration gradient of toxicants or other compounds, which will then produce changes in the monitored impedance.

## 2. Materials and Methods

### 2.1 Fabrication of Au Electrodes

**Fig. 1:**
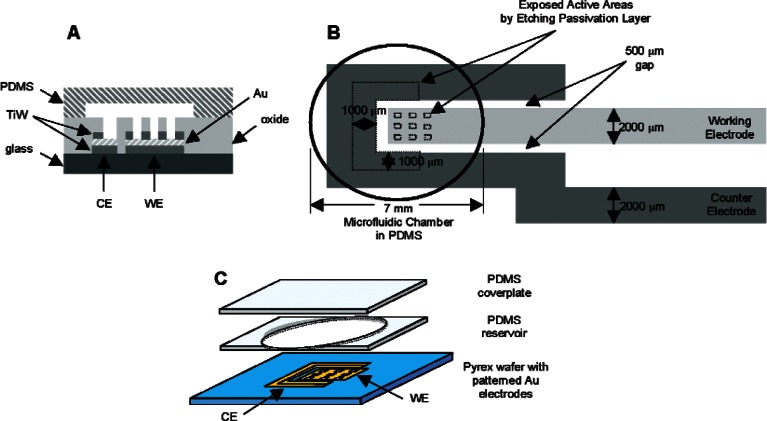
(A) Cross-section of patterned Au electrodes sealed in a PDMS reservoir to form the microfluidic chamber environment. (B) Top-down view of the electrode design consisting of a WE (light gray) surrounded by a CE (dark gray). The active areas of both the CE and WE are denoted with dotted lines. The remaining electrode areas are covered with a SiO_2_ passivation layer. (C) 3-D schematic of fabricated device with a 7 mm-diameter PDMS reservoir surrounding the electrode design. The PDMS reservoir is filled with cell culture medium and sealed with a PDMS coverplate. Drawings not to scale.

The cross-section of the completed device is shown in Fig. 1A. An Au film sandwiched between titanium-tungsten (TiW, Kurt J. Lesker, Co., Pittsburg, PA) adhesion layers is deposited and patterned into a glass substrate, and a silicon dioxide (SiO_2_) passivation layer with openings expose the gold film. A poly(dimethylsiloxane) (PDMS, Sylgard 184, Dow Corning, Midland, MI) reservoir and coverplate are attached and define the microscale cell culture chamber environment. Figure 1B is a top-down drawing of the device showing the working electrode (WE) with an array of patterned openings in the SiO_2_ passivation layer, and a surrounding counter electrode (CE). The cell culture chamber is depicted in Fig. 1B as the area within the circle defined by the PDMS reservoir shown in Fig. 1C.

The electrodes were patterned on 7.62 cm-diameter Pyrex glass wafers (Bullen Ultrasonics, Inc., Eaton, OH). A photolithographic lift-off process was used to pattern the WE and CE onto the wafer. Both electrodes consisted of a 47.5 nm-thick gold layer sandwiched between two 3.0 nm-thick TiW adhesion layers. Next, a 400 nm-thick passivation layer of SiO_2_ was deposited using plasma enhanced chemical vapor deposition (PECVD) at 300°C. The SiO_2_ then underwent reactive ion etching (RIE) to open active electrode areas, which were defined by patterned photoresist. Buffered hydrofluoric acid completed the etching process to remove any trace SiO_2_ remaining on the surface and to expose the underlying TiW layer. The top layer of TiW was etched away from the electrode active areas to expose the underlying gold layer by immersing the wafer in chromium etchant (CR-7) [49].

### 2.2 Fabrication of PDMS Reservoir and Coverplate

A PDMS reservoir was used to form the cell culture chamber environment and was sealed with a PDMS coverplate (Fig. 1C). The 7 mm-diameter reservoir and coverplate were cut out and cleaned with ethanol and deionized water. The reservoir was blown dry with nitrogen gas, aligned, and sealed to surround the electrodes. Cell culture medium and cells were directly seeded into the reservoir before affixing the cover plate on top.

### 2.3 PEM Deposition

Aqueous polyelectrolyte solutions of PEI (Polysciences, Inc., 70 000 g/mol), PSS (Polysciences, Inc., 70 000 g/mol), and PAH (Scientific Polymer Products, Ontario, NY, 70 000 g/mol) were prepared using 18.2 MΩ Milli-Q water (Millipore Corp., Bedford, MA). Sodium chloride (NaCl, Mallinkrodt Baker, Inc., Phillipsburg, NJ) was added to the 1 mmol/L polyelectrolyte solutions to produce a salt concentration of 0.1 mol/L. Altering the ionic concentration of the PEMs solutions provided control of the layer thickness and surface roughness [50]. A low ionic concentration of 0.1 mol/L NaCl produced thin, smooth PEMs layers [25]. The pH levels of the polyelectrolyte solutions were adjusted so that the degree of ionization of the functional groups was appropriate for the formation of the polyelectrolyte layers [27]. This was accomplished by either adding 0.1 mol/L NaOH or 0.1 mol/L HCl until the pH was adjusted to 4.8 to 5.0, 6.0, and 5.0 for PEI, PSS, and PAH, respectively. Any particulates found in solution were removed by filtering the solution with 0.20 m Millex-GN syringe driven nylon filter units (Millipore Corp.).

The PEI polycation was exposed to the gold electrode surface for 25 min to form the initial PEM anchoring monolayer, followed by deionized water rinsing steps to remove any non-bonded PEI. PSS was added to the system for 5 min to form the first polyanion layer. The device was rinsed again, and a PAH polycation layer was added for 5 min and rinsed. Polyelectrolyte multilayers of PSS and PAH were alternately deposited onto the substrate until five PSS/PAH bilayers [(PSS/PAH)_5_] were formed. Previous studies show that five bilayers of PSS/PAH are appropriate to mask the functional groups of the PEI layer, which would otherwise inhibit cell adhesion [51].

### 2.4 Cell Culture

The immortalized NIH-3T3 mouse embryonic fibroblast cell line used in this study was grown in Dulbecco’s Modification of Eagle’s Medium (DMEM, ATTC, Manassas, VA) containing L-glutamine (4 mmol/L), glucose (4500 mg/L), and sodium bicarbonate (1500 mg/L) supplemented with 10 % newborn bovine serum (Invitrogen Corporation, Carlsbad, CA). The cell cultures were incubated at 37°C in 5 % CO_2_. The medium was changed every 2 d, and the cells were allowed to proliferate until approximately 80% confluency was reached.

### 2.5 Cell Density and Surface Area Quantification

Quantifications of NIH-3T3 cell density and average cell surface area during growth on PEM-treated devices were made using optical microscopy with phase contrast, along with ImageJ 1.38x cell counter software plugins (National Institutes of Health, USA) and particle analyzing tools. To check for harmful responses as NIH-3T3 cells grew on the device, the cells were seeded at densities of ≈1.0 × 10^5^ cells/mL (≈4000 cells/cm^2^), which are comparable to those used in a conventional 25 cm^2^ cell culture flask.

### 2.6 Impedance Spectroscopy

**Fig. 2:**
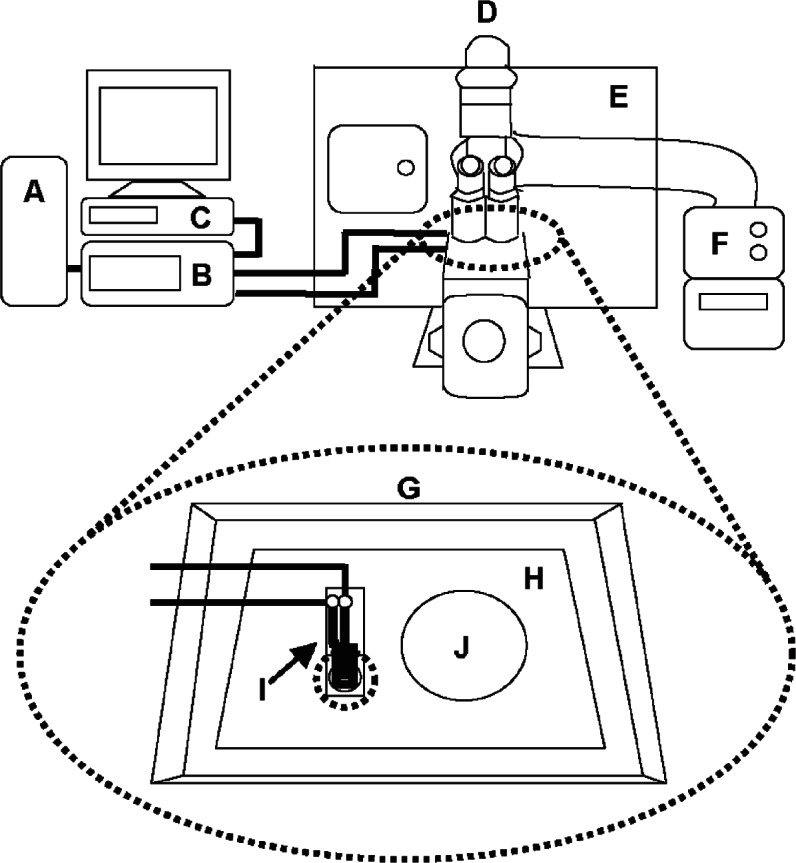
Experimental set-up for cell growth monitoring impedance measurements. The set-up consists of (A) a computer connected to (B) an impedance/gain-phase analyzer with (C) an electrochemical interface. (D) An inverted microscope equipped with (E) a surrounding incubation chamber, as well as (F) a temperature controller, which both help maintain the chamber at 37°C and at a 5% CO_2_ level. The inset image represents a secondary set-up within the incubation chamber. This set-up is enclosed within (G) a microscope stage cover to maintain humidity and CO_2_ levels. It houses (H) a PMMA chip carrier to hold (I) the electrically-connected cell proliferation monitoring device, as well as (J) a small Petri dish containing water to minimize culture medium evaporation. The dotted line located under the device denotes the location of the microscope objective.

Figure 2 illustrates the set-up used for impedance measurements, which utilized a Solartron 1260 Impedance/Gain-Phase Analyzer with a Solartron SI 1287A Electrochemical Interface (Solartron Analytical, Oak Ridge, TN). The AC probe signal used to characterize the impedance was 1 mV peak-to-peak over the frequency range of 1 Hz to 10^6^ Hz. The resulting current never rose above the 1 nA range and, as expected, did not cause any noticeable detrimental effect on the cells [52,53]. Impedance spectra were recorded using the ZPlot2/ZView2 software package (Scribner Associates, Inc., Southern Pines, NC) to measure and record the impedance of the system. Plots of impedance |Z| vs. *f* were used to visualize the raw data and evaluate the quality of data fitting over the entire frequency domain probed.

The humidity (95 % relative humidity), 37°C temperature, and 5% CO_2_ level were all maintained and preserved through the use of an incubation chamber surrounding an Axiovert 200 m inverted microscope (Zeiss, Thornwood, NY). The inset illustration of Figure 2 shows a detector holder consisting of an in-house fabricated poly(methyl methacrylate) (PMMA) chip carrier, which was used to hold the cell confluence detector device in place on the microscope stage. A small Petri dish was included on the detector holder stage to hold ultrafiltrated deionized water to minimize DMEM evaporation. A microscope stage cover surrounded this secondary set-up to maintain the humidity and CO_2_ levels within the confined area.

The cell chamber was filled with warmed and complete DMEM at 37°C. An initial impedance medium control measurement was taken after a 10 min-temperature stabilization period. The microchamber was then seeded with NIH-3T3 cells and covered, and impedance measurements were taken continuously over time in 10 min-intervals until 96 h had elapsed after cell seeding for PEM-treated electrode devices, respectively. Phase contrast images of the cells grown on the device were taken with an AxioCam MRm camera (Zeiss) at t = 0 h, 4 h, 24 h, 48 h, 72 h, and 96 h.

Trypsin [0.25%/ethylenediaminetetraacetic acid (EDTA, 0.53 mmol/L)] in Hank’s buffered salt solution (HBSS) without calcium or magnesium (ATCC) was introduced to the system to promote cell detachment from the electrode surface after confluency was attained at the endpoint of the measurement.

## 3. Results and Discussion

### 3.1 Quantification of Cell Density

**Fig. 3:**
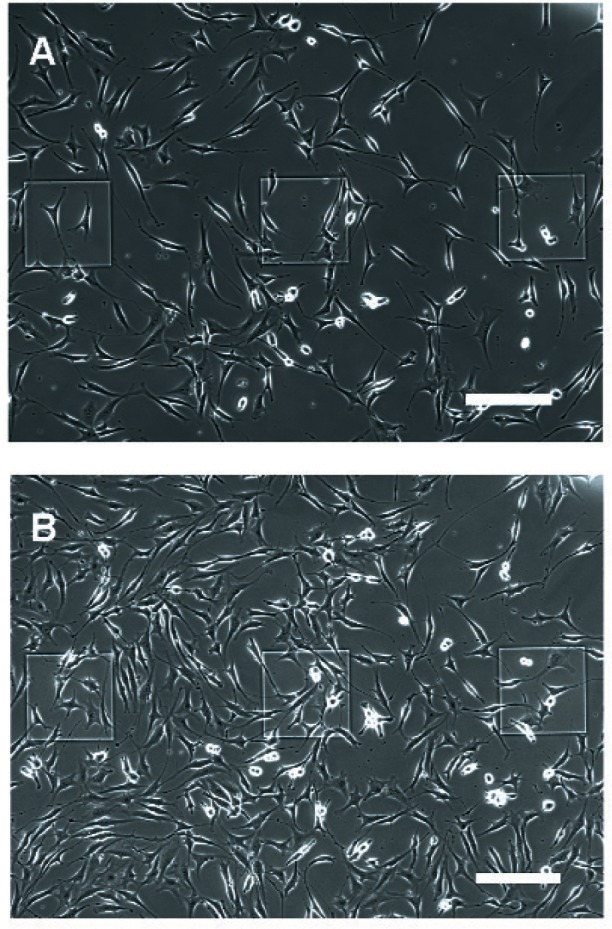
NIH-3T3 fibroblast cells growing on PEM-treated electrodes (A) 48 h and (B) 72 h after cell seeding. When PEMs were deposited onto the electrode surface, the coating covered both the oxide and the bare gold electrode active areas and allowed cell growth to be supported over the entire device surface. All scale bars are 200 µm.

It was desirable that the time for the cells to attain confluency in the 7 mm-diameter growth chamber device be similar to that exhibited by cells grown in a traditional cell culture dish (control). Figure 3 shows NIH-3T3 cells growing on PEM-treated electrodes in the PDMS chambers at (A) 48 h and (B) 72 h after cell seeding. PEI/(PSS/PAH)_5_ covered the electrode active areas, as well as the oxide passivation layer. The figure shows that the PEM treatment effectively hides the existence of the gold surface from the cell culture allowing the cells to propagate uniformly over the surface. Uniform growth over the surface is important when impedance measurements are made on the system, because the measurements will represent a more accurate determination of average cell density on the device.

**Fig. 4:**
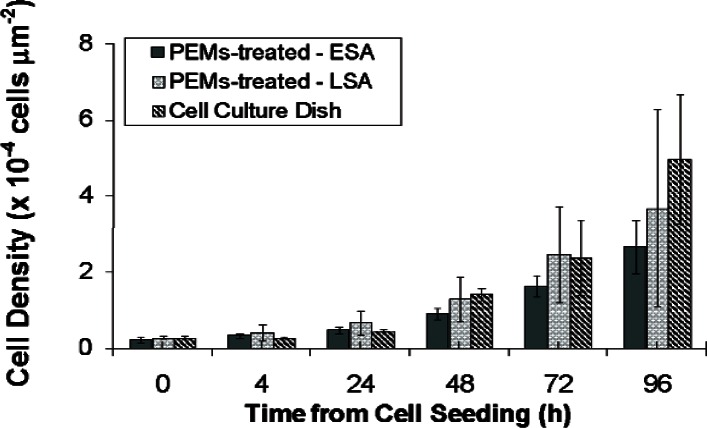
Cell density graphs of NIH-3T3 fibroblast cells growing on PEM-treated and cell culture dish (control) surfaces. Cell growth on PEM-treated devices and in cell culture dishes were discontinued after 96 h. The LSA is restricted to the WE active areas, which have an SA totaling 3.6 ×10^5^ µm^2^. The ESA is total SA of cell culture chamber equaling 1.83 × 0^7^ µm^2^. The two measurements of cell densities on the PEM-treated surfaces closely match the control. This suggests that PEMs are needed to mimic the growth environment of a cell culture dish. Error bars indicate the standard deviation of the average of three cell density measurements.

Figure 4 shows the average change in cell density over time of 3 PEM-treated devices and 3 cell culture dish controls as a function of supportive cell growth area. The experiments were completed on different days over a time span of approximately 3 weeks. The cell density results were collected by distinguishing two types of sampling areas: the localized surface area (LSA) and the entire surface area (ESA). The LSA is defined as the total surface area (SA) of the WE active areas (3.6 ×10^5^ µm^2^) only. The ESA is the complete surface of the cell proliferation monitoring device (1.83 ×10^7^ µm^2^), which includes both the electrode active areas and SiO_2_ passivated areas. The use of separate sampling areas is meant to look for differences between the cell proliferation over the PEM-coated active electrode areas and the PEM-coated oxide layer. When the LSA cell density value is similar to the ESA cell density value, the cells are considered to be dispersed uniformly over the surface, as they would be expected on the cell culture dish controls.

Figure 4 shows both of the PEM-treated devices and the control reaching a final cell density of approximately 2.0 ×10^−4^ cells/µm^2^ at t = 2 h. Therefore, the PEM-treated surfaces allowed normal cell growth to occur at a pace comparable to traditional polystyrene cell culture dishes at equivalent cell seeding densities. For both the LSA and ESA, the cell density calculations were within the error for the cell culture dish control measurements, confirming that the rate of cell growth on PEMs is similar to that on a cell culture polystyrene surface. In addition, because the calculated densities were similar on both LSA and ESA, the cell growth on PEMs can be considered to be growing uniformly across the device surface and electronic monitoring of cell growth over the LSA can be used as a proxy for cell growth over the ESA.

### 3.2 Electronic Measurements of Cellular Proliferation

**Fig. 5:**
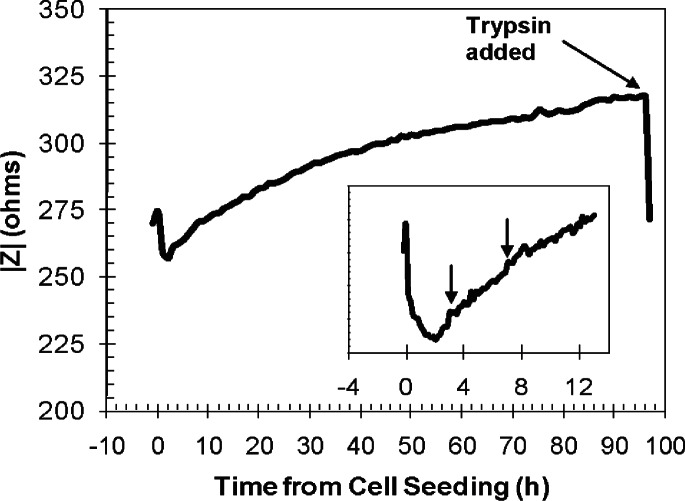
Magnitude of impedance of NIH-3T3 fibroblast cell growth on PEM-treated devices at 1 kHz. The sharp decrease in impedance at the end of the curve denotes the detachment of the cells when trypsin/EDTA is added to the system after continuous impedance monitoring is completed. The inset graph illustrates the change in impedance from cell-free medium until 13 h after cell seeding for a PEM-treated electrode device. Arrows in the inset graph denote areas of constant impedance where cells are flat on the electrode surface and may be moving horizontally across the electrode.

Time-dependent alternating current impedance was used to evaluate the performance of PEM-treated cell-bearing electrodes under conventional cell culture conditions. The previous visual quantification measurements were made in order to interpret the impedance measurements and to be able to quantify cell proliferation by impedance. Figure 5 shows the impedance monitoring of NIH-3T3 cells growing on PEM-treated devices at 1 kHz, the optimal frequency. The medium control measurement is plotted as t = − 1 h from cell seeding. The magnitude of impedance was recorded until 96 h from cell seeding. At the specified endpoint, a trypsin/EDTA solution was added to the system to promote cell detachment, and the impedance was recorded.

In Fig. 5, the cell suspension was added to the system at t = 0 h. There was a decrease in impedance at this time when compared to the cell-free medium measurement at t = − 1 h. The decrease in impedance was an artifact due to the addition of the cell suspension and the adaptation of the cells to the system. The short periods of constant impedance appears to mark the begin ning of cell spreading and proliferation due to cell adhesion [48].

As time passed, the cells moved from being suspended in solution to settling downward towards the electrode surface, where cell attachment and spreading occur. During early time points (inset graph), the small impedance fluctuations may be due to the cells crawling along, as well as moving on and off the electrode surface, (i.e., any horizontal motion) or may also reflect an effect of rounded cells moving from suspension to becoming flat and adhering to the surface. Impedance fluctuations were also apparent at later time points, but cannot be attributed to the previous explanations. These later impedance fluctuations were likely due to the interactions of the cells with the electrode surface, as well as with other cells. Vertical micromotions (i.e., vertical displacements on the order of nanometers, which are significantly beyond the resolution of optical microscopy) of the cells due to changes in cell morphology [2,5] alter the distance of the aqueous gaps between the cell and the electrode surface, thereby affecting the measured impedance detected by the system. Therefore, it is possible that the measured impedance continued to fluctuate even after an approximate steady state was reached because the constant motion of the cells may alter the current flow in subtle ways.

At the specified experimental endpoint for each device type, the cells were subjected to trypsin to promote their detachment from the surface, and a significant drop in impedance was observed (Fig. 5), but the measured impedance did not return to the baseline. This was likely due in part to cleaved adherent proteins or other secreted materials from the cells, as well as other serum components from the culture medium that may have caused biofouling of the PEM coating over the sensing electrodes, thereby offering a resistive contribution to the resulting impedance.

**Fig. 6:**
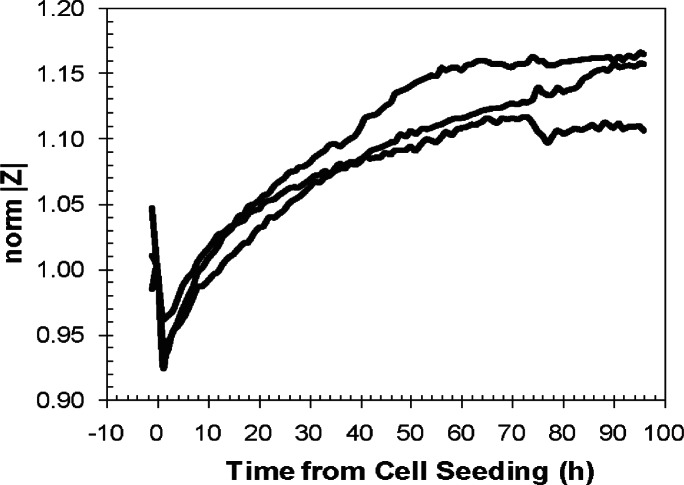
Reproducibility of the normalized magnitude of impedance against t = 0 hours (time of NIH-3T3 fibroblast cell seeding) of PEM-treated devices at 1 kHz performed in triplicate.

The normalized impedance *Z_norm_* (*t*) is defined by Eq. (1):

$$Z_{norm}(t)=\frac{Z(t)}{Z(0)}$$ (1)

where *Z*(*t*) is the measured impedance at time *t*, and *Z*(0) is the measured impedance at the time of cell seeding (t = 0 h). The raw impedance data for NIH-3T3 cell growth on PEM-treated devices was normalized in Fig. 6. The figure shows the graph of the normalized impedance data of 3 different PEM-treated devices.

The increase in normalized impedance was approximately 15 % for the PEM-treated electrode devices. The relatively low change in impedance with cell growth is due to the insulating properties of the polyelectrolyte layers. However, the growth trajectory shown in Fig. 5 appears more than adequate as a proxy for optical cell counts. Overall, the reproducibility of the normalized impedance of the PEM-treated electrodes followed the same trends.

Table 1 reports the mean and standard deviation of the optically-measured number of cells per electrode on the nine PEM-treated electrodes at various times of the three cell-growth experiments shown in Fig. 6. The average number of cells was less than one cell per electrode following seeding (t = 0) for experiments 1 and 3 and the standard deviation was greater than the average in these two cases. Even in experiment 2, where the average number of cells was 2.67, the standard deviation was almost as large as the average. This was likely due to the lack of cell suspension homogeneity at the moment of cell seeding.

**Fig. 7:**
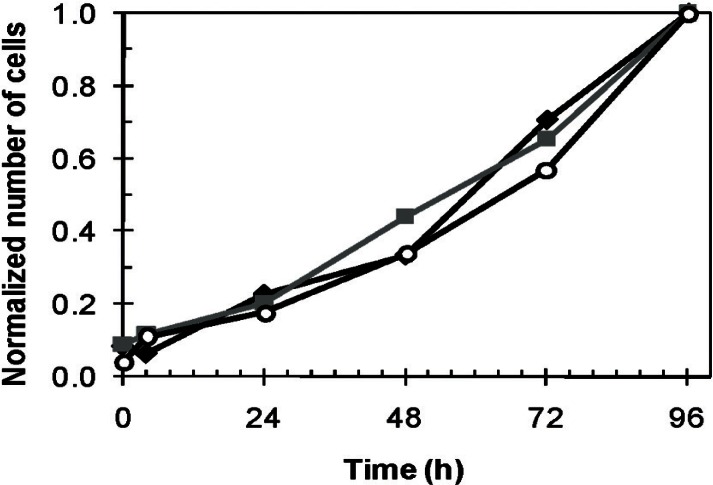
Normalized average number of NIH-3T3 fibroblast cells relative to the cell count at t = 96 h. Cells were counted on each individual active electrode areas (total number of active areas = 9) for three different devices (♦ = 1, **■** = 2, **○** = 3).

The cell number data normalized to the 96-h values are plotted in Fig. 7. First, the shapes of the three growth curves in Fig. 7 are very similar even though the total number of cells at 96 h varied by almost a factor of six for the three experiments. These curves show that cell growth is, while accounting for the uncertainties, approximately exponential. This exponential growth is expected for cells that are proliferating normally, since cells generally double every 18 h to 24 h in culture. For example, the cell growth ratio within a 24-h period (i.e., from Table 1, # cells at t = 48 h/# cells at t = 24 h; # cells at t = 72 h/# cells at t = 48 h; etc.) varied from1.4 to 2.1. The ratio from 0 h to 24 h was not taken into account because cells are not attached onto the surface at t = 0 h. Therefore, only the ratios from 24 h to 96 h were taken into consideration because the cells were already attached on to the surface.

## 4. Conclusions

The fabrication and evaluation of Au electrodes with a surface treatment consisting solely of PEMs to facilitate cell attachment was presented as an impedance-based, real-time cell growth detector. PEM films are an excellent alternative to other biocompatible surface coatings and provide the advantage of a longer biosensor lifetime because of the stable, non-biological nature of the polyelectrolytes from which they are formed. The addition of PEMs promoted cell adhesion and supported normal and uniform cell growth throughout the device surface.

Cellular proliferation measurements utilizing visual inspection in addition to recording electronic impedance measurements were presented. NIH-3T3 fibroblast cell growth followed a normal change in cell density on electrodes modified with PEI/(PSS/PAH)_5_, when compared to polystyrene cell culture dishes. A final cell density of approximately 2.0 × 0^−4^ cells/µm^2^ was observed for both ESA and LSA PEM-treated devices. This value was similar to that on a polystyrene cell culture dish surface at the same confluency level. This suggests that the PEM-treated surfaces promote comparable cell growth responses as traditional polystyrene cell culture surfaces.

The trends of the impedance measurements followed the adhesion, proliferation, and growth of NIH-3T3 fibroblast cells. The magnitude of impedance increased as the number of cells on the surface of the electrode increased, and the measured impedance on the PEM-treated electrodes represented the average proliferation over the entire device surface area. Normalized impedance data showed that the devices reached an approximate steady state value. The PEM-treated electrodes were sensitive enough to probe cell attachment and proliferation while being able to provide a stable and reproducible biocompatible coating for electrode surfaces. In addition, the normalized impedance measurements, indicated that the PEM-treated electrode device is more sensitive during the first 2 to 3 days of cell growth in culture and thus could be more in proliferation measurements during that period. The measurements of the normalized number of cells on individual active electrode areas (LSA) showed that the cell proliferation rate on these areas is occurring at about the same rate seen in traditional polystyrene culture dishes. Furthermore, when comparing the cell proliferation rate on the active areas (LSA) with the rate on the entire surface area of the device (ESA) it was observed that both proliferation rates were similar. Therefore, it appears desirable to use PEMs as a biocompatible electrode surface coating to allow the resulting impedance measurements to be an accurate gauge of average cell growth over the entire surface of the cell proliferation-monitoring device, regardless of the sampling size (ESA vs. LSA). This demonstration validates the use of a PEM-treated cell growth monitoring device for the future application of electronically measuring cell responses to varying concentrations of environmental stimuli (i.e., cytotoxins) by using an array of small, independently-addressable electrodes within microfluidic devices.

## Figures and Tables

**Table 1:** Mean number of cells (µ) located on an individual active electrode area and the standard deviation of the mean (σ) at the nine active area positions for 3 cell growth experiments

Time (h)	µ_1_	σ_1_	µ_2_	σ_2_	µ_3_	σ_3_
0	0.444	0.726	2.67	2.55	0.667	1.32
4	0.333	0.500	3.56	2.92	1.78	1.86
24	1.22	1.20	6.11	5.04	2.89	1.45
48	1.78	1.39	13.2	10.1	5.56	2.40
72	378	1.64	19.7	13.0	9.33	3.39
96	5.33	2.74	30.0	13.2	16.3	7.38

## Footnotes

1 Certain commercial equipment, instruments, or materials are identified in this paper to foster understanding. Such identification does not imply recommendation or endorsement by the National Institute of Standards and Technology, nor does it imply that the materials or equipment identified are necessarily the best available for the purpose.
